# Hypoglycemia-Associated EEG Changes in Prepubertal Children With Type 1 Diabetes

**DOI:** 10.1177/1932296816634357

**Published:** 2016-02-25

**Authors:** Grith Lærkholm Hansen, Pia Foli-Andersen, Siri Fredheim, Claus Juhl, Line Sofie Remvig, Martin H. Rose, Ivana Rosenzweig, Sándor Beniczky, Birthe Olsen, Kasper Pilgaard, Jesper Johannesen

**Affiliations:** 1 Pediatric Department, Copenhagen University Hospital Herlev, Denmark; 2 Hypo-Safe A/S, Lyngby, Denmark; 3 Department of Medicine, Hospital of South West Denmark, Esbjerg, Denmark; 4 Sleep and Brain Plasticity Centre, Department of Neuroimaging, King’s College London, London, UK; 5 Sleep Disorders Centre, Guy’s and St Thomas’s Hospitals NHS Trust, London, UK; 6 Department of Clinical Neurophysiology, Danish Epilepsy Center, Dianalund, Denmark; 7 Aarhus University, Århus, Denmark; 8 Pediatric Department, Copenhagen University Hospital, Hillerød, Denmark; 9 Faculty of Health and Medical Sciences, Copenhagen University, Denmark

**Keywords:** children, diabetes, electroencephalogram, hypoglycemia

## Abstract

**Background:**

The purpose of this study was to explore the possible difference in the electroencephalogram (EEG) pattern between euglycemia and hypoglycemia in children with type 1 diabetes (T1D) during daytime and during sleep. The aim is to develop a hypoglycemia alarm based on continuous EEG measurement and real-time signal processing.

**Method:**

Eight T1D patients aged 6-12 years were included. A hyperinsulinemic hypoglycemic clamp was performed to induce hypoglycemia both during daytime and during sleep. Continuous EEG monitoring was performed. For each patient, quantitative EEG (qEEG) measures were calculated. A within-patient analysis was conducted comparing hypoglycemia versus euglycemia changes in the qEEG. The nonparametric Wilcoxon signed rank test was performed. A real-time analyzing algorithm developed for adults was applied.

**Results:**

The qEEG showed significant differences in specific bands comparing hypoglycemia to euglycemia both during daytime and during sleep. In daytime the EEG-based algorithm identified hypoglycemia in all children on average at a blood glucose (BG) level of 2.5 ± 0.5 mmol/l and 18.4 (ranging from 0 to 55) minutes prior to blood glucose nadir. During sleep the nighttime algorithm did not perform.

**Conclusions:**

We found significant differences in the qEEG in euglycemia and hypoglycemia both during daytime and during sleep. The algorithm developed for adults detected hypoglycemia in all children during daytime. The algorithm had too many false alarms during the night because it was more sensitive to deep sleep EEG patterns than hypoglycemia-related EEG changes. An algorithm for nighttime EEG is needed for accurate detection of nocturnal hypoglycemic episodes in children. This study indicates that a hypoglycemia alarm may be developed using real-time continuous EEG monitoring.

Hypoglycemia is the most frequent acute complication in children with type 1 diabetes (T1D). Despite results from contemporary cohorts of children with T1D no association between severe hypoglycemia and metabolic control can be demonstrated^1-3^ in contrast to previous findings,^4^ this new information still has to be accepted by patients and their parents.

Parents of children with type 1 diabetes report considerable parental anxiety related to the possible occurrence of hypoglycemia, affecting both parental and child health and quality of life.^5,6^ It is suggested that hypoglycemia avoidance behavior of the parents might adversely affect glycemic control of their offspring.^5^ In children, severe hypoglycemic events may arise due to lack of awareness of symptoms or inability to react appropriately at the time of warning symptoms. Impaired awareness may affect up to approximately one-third of pediatric patients with diabetes.^7,8^

Normal brain function depends on continuous glucose supply and it is well described that hypoglycemia is associated with changes in the electroencephalogram (EEG), namely increased activity in the low frequency bands.^9,10^ In a previous study in adults, EEG remained unaffected with blood glucose levels above 3 mmol/l, but following a gradual decline in blood glucose the EEG changes became apparent in all patients. At a median blood glucose concentration of 2.0 mmol/l, the alpha activity (8-12 Hz) decreased while the theta activity (4-8 Hz) increased. Normal EEG was reestablished when the blood glucose concentration exceeded 2.0 mmol/l.^10^

It has previously been demonstrated that hypoglycemia associated EEG changes in adults recorded by subcutaneously placed electrodes precede cognitive failure both during daytime and during sleep.^11,12^ The aim is to develop a hypoglycemia alarm based on continuous EEG measurement and real-time signal processing. However, differentiation of hypoglycemic EEG patterns from normal activity is significantly more challenging in children, since their normal, on-going (“background”) EEG activity contains activity in the slow frequency range,^13^ similar to the ones triggered by hypoglycemia. In addition, the high proportion of slow (delta and theta) activity during sleep makes detection even more difficult during that period. Furthermore, sleep related EEG is known to change dramatically from adolescence to adulthood with an approximately 40% reduction in slow wave sleep.^14,15^

The purpose of this study was to explore the possible difference in the EEG pattern between euglycemia and hypoglycemia in children with T1D during daytime and during sleep. The secondary goal was to trace early warning events of hypoglycemia in children by continuous EEG monitoring.

## Material and Methods

The study was approved by the local Ethics Committee and was performed in accordance with the Helsinki declaration. Study participants and the parental authorities were given written and verbal information before the first visit and signed informed consent.

Eight T1D patients aged 6-12 years completed a series of 3 studies at 3 separate days: (1) daytime induced hypoglycemia, (2) nocturnal normoglycemia, and (3) nocturnal induced hypoglycemia. Only the data from the 2 experiments with induced hypoglycemia are analyzed in this study. Inclusion criteria were: a minimum duration of diabetes of 2 years, age at inclusion of 6-12 years, and a minimum of 2 episodes of nocturnal hypoglycemia within the last year, defined as either biochemical hypoglycemia (blood glucose < 2.5 mmol/l) or symptomatic hypoglycemia. Daily treatment regimens with insulin injections (basal-bolus) as well as continuous subcutaneous insulin infusion (CSII) were accepted. Exclusion criteria were: anemia, abnormal electrocardiogram (ECG) or any other additional medical disease requiring medication, including epilepsy or any previous episode of hypoglycemia-induced seizure. None of the participants had experienced severe hypoglycemia (defined as seizure or unconsciousness) in the year preceding the study.

Demographics of the study participants are detailed in Table 1.

**Table 1.** Demographics of the Study Participants (N = 8).

Participant characteristics	
Gender (boys/girls)	4/4
Age (years)	10.3 [6.4-12.5]
BMI (kg^-1^ m^2^)	17.1 ± 1.4
Diabetes duration (years)	2.7 [1.1-5.7]
Insulin dose (U/kg/d.)	0.74 ± 0.19
Insulin treatment (pen (basal/bolus)/CSII)	1/7
HbA1c (IFCC, mmol/mol)	55 ± 1
HbA1c (NGSP, %)	7.2 ± 0.1
Plasma glucose at nadir, day (mmol/L)	2.3 ± 0.2
Plasma glucose at nadir, night (mmol/L)	2.1 ± 0.1

BMI and insulin dose are expressed as mean ± SD. Age and diabetes duration are expressed as median [range]. Remaining continuous data are expressed as mean ± SEM.

Study procedures were as outlined below. Continuous EEG monitoring was performed throughout all 3 sessions.

All participants were informed that hypoglycemia should be avoided 2 weeks prior to the testing which they by self-reporting confirmed at the study days. During the 48 hours preceding the studies none of the participants experienced BG < 4,5 mmol/l (3 of the 8 participants had a continuous glucose monitor and the remaining all had a routine of measuring BG minimum 5 times daily at specific times).

### Hyperinsulinemic Hypoglycemic Clamp (Induced Hypoglycemia)

#### Preparation

##### Daytime

The patients met fasting in the clinical research unit, and no insulin was injected in the morning (in case of insulin pump therapy the pump was disconnected).

##### Nighttime

Patients were instructed to take their normal insulin dose during the daytime. No long acting insulin was administered during the evening (in case of insulin pump therapy the pump was disconnected). The clamp was initiated when the participant was asleep determined by EEG changes.

In the clinical research unit, blood glucose was measured every 30 minutes until start of the clamp while the monitoring equipment was attached. EEG and ECG were continuously monitored. In addition electromyogram (EMG) and electrooculogram (EOG) electrodes were applied during induced hypoglycemia at night.

A polyethylene catheter was placed bilaterally in an antecubital vein for test infusions and venous blood sampling.

### Hyperinsulinemic Clamp Procedure

Continuous high dose insulin infusion (80 mUI/m^2^/min, NovoRapid, NovoNordisk A/S, Bagsværd, Denmark) together with continuous glucose infusion (20%) was initiated. Venous glucose concentration was maintained at 5-7 mmol/l. Glucose infusion rates were adjusted every 5 min according to bed-site glucose measurements. Following 45 min of euglycemia a gradual decrease in glucose infusion was performed. The hypoglycemia was ceased (1) when plasma glucose was lower than 2.2 mmol/l at 2 consecutive measurements, (2) if the patient was obviously cognitively impaired by the low blood glucose level, or (3) at the demand of the patient/parent or (iv) by the investigator. Restoration of euglycemia was obtained by ending insulin infusion and increasing the glucose infusion rate while allowing the patient to eat and drink. Venous glucose was measured every 5 minutes on an ABL glucose monitor until normoglycemia was reached and stabilized (BG> 6 mmol/l). The patient could leave the clinic after 2 hours observation or the following morning.

EEG electrodes were placed as detailed below.

### Nocturnal Normoglycemia

To assure no recent hypoglycemic events and to make the child comfortable sleeping in a clinical setting, a nocturnal observation was performed the night prior to the third experiment (nocturnal induced hypoglycemia). A polyethylene catheter was placed in an antecubital vein and blood glucose measured every 30 min. EEG electrodes were placed as detailed below.

### EEG Monitoring

**Figure 1.**
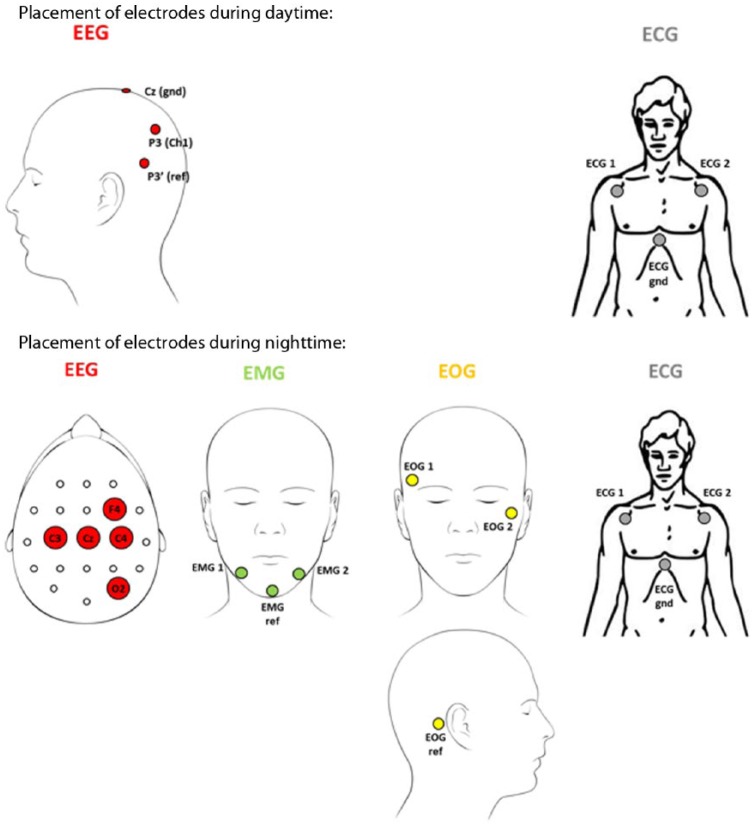
Electrode placements.

Continuous EEG monitoring was recorded using standard scalp electrodes (reusable silver EEG cup electrodes, 200 cm leads, 10 mm cup, Embla Systems Inc, Amsterdam, Netherlands). The position of the electrodes was different for the daytime and nighttime experiments (see Figure 1). During daytime, the EEG electrodes was positioned at P3 with the reference electrode positioned 4 cm below in the direction of T5. Automatic detection of hypoglycemia associated EEG changes has previously been demonstrated for this electrode position.^11^ For the nocturnal experiments, the EEG electrodes were positioned at C4-O2. The C4-O2 location is considered suitable for detection of the hypoglycemia EEG paradigm and enables for sleep analysis. This different location does not affect the qEEG analysis. The recorded EEG signals were sampled with a g.USBamp (Guger Technologies, Graz, Austria) at 64 Hz and preprocessed using a digital Butterworth 8th order 0.5 to 30 Hz band-pass filter.

Sleep scoring was performed by 2 experts (IR and SB) according to the international standards.^15^ The 2 experts were blinded for the blood glucose values.

### Data Extraction for qEEG Analysis

#### Daytime

Thirty minutes of consecutive EEG from the beginning of the experiment (plasma glucose >5.0 mmol/l) represent the euglycemic state, while 5 minutes of consecutive EEG at the glycemic nadir represent the hypoglycemic state. The 30 minutes were chosen as the best (less noisy) consecutive data sequence.

#### Nighttime

The nocturnal EEG characteristics are highly dependent on the present sleep stage. Therefore, the euglycemic EEG was partitioned into sleep stage dependent sequences, each of which was compared to the hypoglycemic EEG. Only data before the hypoglycemic event were included. For some experiments, the amount of data before the hypoglycemic event was sparse. Therefore, a limit of 3.5 mmol/l was chosen rather than 5.0 mmol/l (as for daytime) to include a sufficient amount of data representing the euglycemic state.

The hypoglycemic state was represented by 5 min of consecutive EEG at the glycemic nadir as for the daytime data.

### qEEG Analysis

For each subject, quantitative electroencephalogram (qEEG) measures were calculated from both euglycemic and hypoglycemic (day and night) EEG. Based on visual inspection of the overall EEG patterns, the EEG signal quality was considered good.

For each subject, a median power spectral density of the euglycemic and hypoglycemic sequences was estimated using a modified Welch’s method that determines the median spectrum rather than the average spectrum. The following settings were applied for the power spectral density estimation: 4 s Hamming-windowed epochs, 50% overlap, 0.25 Hz resolution. In the nighttime analysis, a euglycemic spectrum was estimated for each sleep stage (see later). From the power spectral densities, median amplitude spectra were calculated (from the square root of the power), each of which was then subdivided into 3 traditional frequency bands: delta (1-3.75 Hz), theta (4-7.75 Hz), and alpha (8-12.75 Hz). In addition, a unified theta:alpha band was implemented (4-12.75 Hz), since a dominant peak in the alpha band may be transferred to the theta band by just a slight slowing of the frequency. Likewise, a unified delta:theta band (1-7.75 Hz) was introduced for the night time analysis. For each band, the absolute amplitude and the centroid frequency were calculated. The absolute amplitude was determined using a numerical integration technique applying the trapezoidal rule. The centroid frequency was defined as the center of gravity of each frequency band that subdivides the area under the spectral curve into 2 of equal size.

Signal processing was conducted in Matlab 7.12.0 (R2011a).

### Statistical Analysis

Spectral EEG characteristics differ across subjects, subsequently we conducted within-patient analysis comparing hypoglycemia versus normoglycemia changes in each qEEG measure.

The nonparametric Wilcoxon signed rank test for zero medians was performed. In particular, a 2-sided signed rank test of the hypothesis that the difference between the paired eu- and hypoglycemic qEEG measure comes from a distribution whose median is zero. The nonparametric test was chosen due to the small population size of 8 subjects and because the EEG characteristics change remarkably depending on age.

### The EEG-Based Hypoglycemia Detection Algorithm

Two distinct EEG-based hypoglycemia detection algorithms have been developed for adults: 1 for use during daytime analyzing awake EEG^11^ and 1 for nocturnal EEG.^12^ A dedicated algorithm for nighttime use is required due to the deep sleep slow wave EEG patterns, which resemble the hypoglycemia-related EEG changes.

In this study, the EEG-based hypoglycemia detection algorithm developed for adults were tested on prepubertal children. For the daytime algorithm, a leave-one-out threshold adjustment was performed to adjust the algorithm for the recorded prepubertal EEG. For the nighttime algorithm, this was not possible due to excessive false detection rates during deep sleep EEG.

## Results

### Daytime qEEG Results

Table 2 lists the results of the daytime analyses. An increase in the absolute amplitude of the delta, theta, alpha, and theta:alpha band is present during hypoglycemia compared to euglycemia. In addition, an alpha centroid frequency decrease is present during hypoglycemia.

**Table 2.** Results daytime (N = 8).

	Absolute amplitude, hypo-normo change				Centroid frequency, hypo-normo change			
	Median	Range		*P* value	Median	Range		*P* value
Delta	**3.61**	**0.93**	**9.45**	**.008**	0.03	−0.09	0.24	.25
Theta	**2.50**	**0.96**	**14.80**	**.008**	0.01	−0,11	0.07	.844
Alpha	**1.71**	**1.11**	**3.50**	**.008**	**−0.04**	**−0.25**	**0.02**	**.039**
Theta:alpha	**4.82**	**2.12**	**18.44**	**.008**	−0.07	−0.73	0.25	.313

Medians, ranges, and *P* values of paired hypoglycemia versus normoglycemia changes. A positive change means that the qEEG measure value in the hypoglycemic state exceeds that of the euglycemic state. Values in bold: P < 0.05.

### Nighttime qEEG Results

Table 3 lists the results of the nocturnal experiments. As the EEG patterns show alterations dependent on the sleep stage, data within the different sleep stages during euglycemia were extracted. The data set extracted for analysis was complete for sleep stage 3 (n = 8 participants, mean 65.5 minutes [17-133]), insufficient for REM sleep (n = 2) and nearly complete for sleep stage 1 (n = 7, mean 7.9 minutes [3-15]) and for sleep stage 2 (n = 7, mean 36.0 minutes [6-85]).

**Table 3.** Results Nighttime: *P* values, Medians, and Ranges of Paired Changes: Hypoglycemia (at Sleep) Versus Normoglycemia (at Awake and Sleep Stages N1-N3). A.

	Absolute amplitude, hypo-normo change				Centroid frequency, hypo-normo change			
Awake (n = 7)	Median	Range		*P* value	Median	Range		*P* value
Delta	**13.08**	**3.04**	**33.06**	**.016**	−0.07	−0.18	0.08	.109
Theta	**5.37**	**0.92**	**13.83**	**.016**	0	−0.13	0.06	.813
Alpha	0.13	−4.27	7.73	.688	0.02	−0.38	0.23	.813
Delta:theta	**18.69**	**5.48**	**47.86**	**.016**	**−0.28**	**−0.42**	**0.08**	**.031**
Theta:alpha	**4.89**	**−3.34**	**15.51**	**.031**	**−0.39**	**−0.9**	**0.05**	**.031**

**Table:** B.

	Absolute amplitude, hypo-normo change				Centroid frequency, hypo-normo change			
N1 (n = 7)	Median	Range		*P* value	Median	Range		*P* value
Delta	**9.38**	**3.17**	**31.3**	**.016**	**−0.09**	**−0.19**	**0.05**	**.031**
Theta	2.26	−2.24	12.41	.156	0.07	−0.1	0.16	.375
Alpha	2.24	−2.08	8.53	.109	0.05	−0.48	0.15	1
Delta:theta	**10.79**	**0.88**	**44.6**	**.016**	**−0.27**	**−0.42**	**0.09**	**.031**
Theta:alpha	8.11	−3.92	14.91	.156	0.23	−0.53	0.57	.375

**Table:** C.

	Absolute amplitude, hypo-normo change				Centroid frequency, hypo-normo change			
N2 (n = 7)	Median	Range		*P* value	Median	Range		*P* value
Delta	3.3	−0.47	26.41	.078	**−0.09**	**−0.16**	**0.05**	**.047**
Theta	0.63	−2.8	10.41	.469	0.03	−0.03	0.15	.078
Alpha	0.7	−0.36	6.99	.078	−0.03	−0.6	0.14	.375
Delta:theta	3.6	−3.46	37.58	.078	−0.17	−0.25	0.15	.078
Theta:alpha	5.7	−3.13	10.95	.109	0.23	−0.7	0.59	.375

**Table:** D.

	Absolute amplitude, hypo-normo change				Centroid frequency, hypo-normo change			
N3 (n = 8)	Median	Range		*P* value	Median	Range		*P* value
Delta	**−26.56**	**−33.4**	**8.38**	**.039**	0.04	−0.05	0.18	.148
Theta	−4.87	−12.08	3.79	.055	0.03	−0.07	0.19	.313
Alpha	−0.35	−3.4	6.76	.945	−0.02	−0.32	0.17	.945
Delta:theta	−33.79	−46.57	12.22	.055	0.14	−0.1	0.61	.055
Theta:alpha	−1.87	−13.63	3.26	.195	0.18	−0.26	1.06	.148

A positive change means that the qEEG measure value in the hypoglycemic state exceeds that of the euglycemic state. Values in bold: P < 0.05.

Comparing hypoglycemia during sleep to awake state normoglycemia (at night before falling asleep) (Table 3A) showed a significant increase in the absolute amplitude of the delta, theta, delta:theta and theta:alpha bands and a decrease in the centroid frequency of the delta:theta and theta:alpha bands.

When pairing the hypoglycemic state versus normoglycemia (during sleep) in sleep stages I-III we saw changes primarily in the delta band.

In sleep stage I (Table 3B) we found an increase in the absolute amplitude of both the delta and the delta:theta bands. Furthermore, a significant decrease in the centroid frequencies of the delta and delta:theta bands was found. In sleep stage II (Table 3C) there was a significant, although slight decrease in the centroid frequency of the delta band. In sleep stage III (Table 3D) we found a reduction in the absolute amplitude of the delta band.

As mentioned earlier the data set for REM sleep was not sufficient.

### Sleep

According to the scoring based on EEG, EOG and EMG, the sleep efficiency was 86% and 90% during the control night and the night of induced hypoglycemia, respectively. During the night of induced hypoglycemia the subjects spent 6% of the nighttime in sleep stage N1, 36% in sleep stage N2, 33% in sleep stage N3, and 16% in REM sleep. All subjects were clinically sleeping prior to the induction of hypoglycemia. The sleep stage during the last 5 minutes prior to hypoglycemia nadir was N3 for 5 subjects, W+N1+N2 for 1 subject, W+N2+N3 for another, and N2+N3 for 1 subject.

### Algorithms

#### Daytime

The automated EEG-based day time algorithm detected the induced hypoglycemia in all children on average at BG of 2.5 ± 0.5 mmol/l (range 1.7-3.0 mmol/l) and 18.4 ± 20.3 minutes (range 0-55 minutes) prior to blood glucose nadir on average 2.3 ± 0.5 mmol/l. In none of the cases the algorithm indicated hypoglycemia at BG above 3.5 mmol/l.

#### Nighttime

The automated EEG-based nighttime algorithm did not perform. It was more sensitive to deep sleep EEG patterns than hypoglycemia-related EEG changes.

## Discussion

To the best of our knowledge, the present study is the first to examine EEG changes and testing an EEG-based prediction algorithm during induced hypoglycemia both during daytime and during sleep in prepubertal children.

We demonstrate significant differences within the quantitative EEG recorded during euglycemia and hypoglycemia obtained during awake state as well as during sleep. The real-time analyzing algorithm developed for adults detected accurately hypoglycemic events in children during the awake state. However, due to the high proportion of slowing occurring during sleep in children, the daytime algorithm had too many false alarms when applied on nighttime data.

During induced hypoglycemia in daytime we found a significant increase in the absolute amplitude of the delta, theta, alpha, and theta:alpha band compared to euglycemia. These findings correspond well with earlier studies in both adults and children showing increased activity in the low-frequency bands during hypoglycemia.^16-19^ In addition, a decrease in the alpha centroid frequency was present during hypoglycemia in consistence with findings from adult studies.^16^

The interpretation of the nocturnal EEG is difficult since the EEG during sleep is characterized by occurrence of slow wave patterns which is also seen during hypoglycemia. It has earlier been shown that it is possible to differentiate the characteristic EEG sleep pattern from that of hypoglycemia in adults.^12^ However, the sleep distribution pattern and the EEG pattern in children both undergo changes during adolescence and differ in several ways from the adult patterns. Despite this, we were able to detect significant changes during induced hypoglycemia at sleep although limited to the delta and delta:theta band. Algorithms specifically targeting these features are needed for accurate detection of nocturnal hypoglycemic episodes in children.

The present study was designed to compare the hypoglycemia EEG pattern at sleep to the various sleep EEG patterns during euglycemia, hence 1 hypoglycemia pattern at sleep versus the various sleep stages in euglycemia. Ideally, one might compare the hypoglycemia pattern obtained in, for example, N1 versus N1 euglycemia, and so on, however such a design is almost impossible to obtain.

Few studies have investigated EEG changes during nocturnal hypoglycemia in adolescents.^20-22^ Partly in line with our findings, the study of Nguyen and Jones^20^ of 6 patients with T1D demonstrated a reduction in the centroid alpha frequency and an increase in the centroid theta frequency associated with hypoglycemic episodes at night. In Nguyen et al^21^ and Nguyen et al^22^ 5 patients were studied. These studies apply different analytical methodologies to the EEG data obtained and both demonstrated qualitative differences in the EEG pattern between sleep in normoglycemia and hypoglycemia. Within the latter article, changes between the hypoglycemic phase and the recovery phase were also demonstrated. However, all 3 studies included only adolescents and did not describe EEG changes correlated to the different sleep stages nor sought to establish real-time EEG analysis.

In our study, the EEG-based daytime algorithm identified hypoglycemia in all children on average at BG of 2.5 ± 0.5 mmol/l and 18.4 (ranging from 0 to 55) minutes prior to blood glucose nadir. The previously published hypoglycemia detection algorithm developed in adult diabetes subjects^11^ was unable to distinguish between episodes of deep sleep and episodes of hypoglycemia. Further investigation, using a novel algorithm specifically targeting the quantitative changes observed in children during the night, is needed to develop a functional algorithm for detecting hypoglycemia during sleep.

## Conclusions

We found quantitative EEG features distinguishing between euglycemic and hypoglycemic states in children, both daytime and during sleep. An automated algorithm accurately detected hypoglycemic episodes during daytime. However, during the night the algorithm gave too many false positives. This indicates that a hypoglycemia alarm may be developed using real-time continuous EEG monitoring. Our study is limited by the number of participants and further experiments are obviously needed to confirm the reproducibility of the EEG characteristics, in particular during sleep.
